# Exploration of Copper Halide Linear‐Array Detector Prototype for Security Checks

**DOI:** 10.1002/smll.202514814

**Published:** 2026-02-05

**Authors:** Yang Zhou, Tengyue He, Wenyi Shao, Wentao Wu, Peng Yuan, Haijiao Xie, Osman M. Bakr, Omar F. Mohammed

**Affiliations:** ^1^ Center for Renewable Energy and Storage Technologies (CREST) Division of Physical Science and Engineering King Abdullah University of Science and Technology Thuwal Kingdom of Saudi Arabia; ^2^ School of Materials Science and Engineering University of Jinan Jinan Shandong China; ^3^ Hangzhou Yanqu Information Technology Co., Ltd. Xixi Legu Creative Pioneering Park Hangzhou Zhejiang China

**Keywords:** copper halide, linear‐array detector, scintillator, X‐ray imaging

## Abstract

The structural tunability and photophysical richness of metal halides make them ideal for exploring next‐generation X‐ray scintillators. Although numerous candidates have been reported in recent years, their commercial viability remains to be rigorously demonstrated. Among them, the high‐density all‐inorganic low‐dimensional copper halide Cs_3_Cu_2_I_5_ with a soft lattice and pronounced electron–phonon coupling, readily generates a self‐trapped exciton and thus delivers efficient, self‐absorption‐free emission. In this work, we further modified the scintillation performance of Cs_3_Cu_2_I_5_ by Mn^2^^+^ incorporation. The optimized composition delivers 1.35 times the relative light output of CsI: Tl, with a detection limit down to 33.1 nGy s^−1^. Besides, the copper halide scintillator possesses negligible afterglow and a fast X‐ray excitation decay time of 46.4 µs. More importantly, the copper halide scintillator exhibits exceptional light output, which is ∼18% higher than that of a commercial imaging scintillator (Carestream Min‐R 2190), through detector‐level determination. We demonstrate a previously unexplored implementation of a powder‐based copper halide scintillator in a linear‐array detector, achieving a spatial resolution of 1.1 lp/mm and showing strong potential for enhanced security inspection applications.

## Graphical abstract

Mn‐doped Cs_3_Cu_2_I_5_ is demonstrated as a highly promising scintillator for X‐ray imaging, featuring high light yield, a low detection limit, and negligible afterglow. A linear‐array detector prototype based on the Mn‐doped Cs_3_Cu_2_I_5_ delivers a spatial resolution of 1.1 lp/mm in food‐safety and security inspections, marking a significant advance in the practical application of metal halide scintillators.

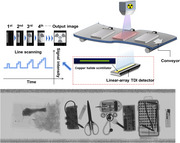

## 1 Introduction

**SCHEME 1:**
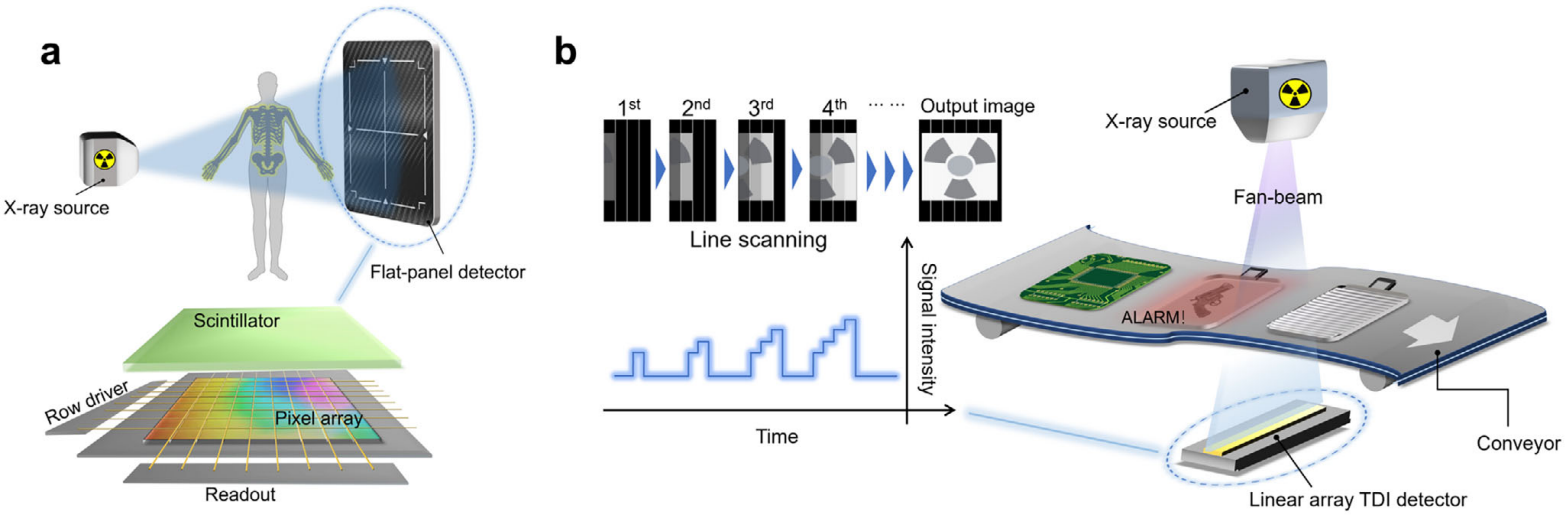
Schematic diagrams of scintillator‐mediated indirect X‐ray detectors with two different scanning modes: (a) flat‐panel X‐ray detector and (b) TDI linear‐array X‐ray detector.

Scintillator‐mediated X‐ray detectors are critical for applications in medical diagnostics, as well as industrial and security inspections [1, 2, 3, 4, 5]. Currently, scintillator‐mediated X‐ray detectors can be classified into flat‐panel and linear‐array detectors based on the configuration of sensor array pixels to meet the demands of different scenarios [6, 7, 8, 9]. The sensor pixels of a flat‐panel detector are organized in a regular 2D array, allowing for the generation of 2D projected images of static or dynamic objects within a finite field of view. This is achieved through plane scanning using single or multiple frames, as shown in Scheme 1a. In contrast, the linear‐array detector consists of sensors organized in a 1D linear configuration, facilitating line scanning of objects moving perpendicular to the pixel rows. The images captured from each pixel row are sequentially stitched and read out, enabling effective monitoring of large objects in uniform motion. Notably, the time delay integration (TDI) linear‐array detector distinguishes itself from conventional linear‐array scanners, which typically utilize one or a few rows of pixels. TDI detectors consist of tens to hundreds of pixel rows. During image acquisition, signals generated by each pixel row are rapidly read and integrated with the subsequent row, accumulating sequentially over time until all signals are collectively read out (Scheme 1b). This TDI technology enables linear‐array detectors to capture significantly more photons per unit exposure time by aggregating signals across multiple pixels. As a result, TDI detectors provide a higher signal‐to‐noise ratio, improved scanning speed, and a broader dynamic range. These advancements are particularly crucial for detecting hard‐to‐penetrate objects in security and quality inspections.

On the other hand, an ideal scintillator is essential for X‐ray detectors to reduce radiation dose and enhance spatial resolution in imaging [1, 10]. After over a century of progress, CsI: Tl and Gd_2_O_2_S: Tb have dominated the field of X‐ray imaging scintillators [11, 12]. However, their high manufacturing demands and costs, along with long afterglow and decay times, remain critical factors driving the development of novel scintillation materials [1]. Metal halides with diverse chemical structures and photophysical properties have emerged as an ideal material system for exploring new X‐ray imaging scintillators [13, 14, 15, 16, 17, 18, 19]. High‐density all‐inorganic low‐dimensional copper halides, characterized by their soft lattice and pronounced electron‐phonon coupling, readily form a self‐trapped exciton (STE), which enables efficient luminescence without re‐absorption [20, 21, 22, 23, 24]. Our previous work demonstrated that a large‐area, flexible Cs_3_Cu_2_I_5_ film realized a high spatial resolution of 17 lp/mm using a homemade X‐ray imaging setup [25]. Moreover, introducing trace amounts of additional metal element activators (e.g., manganese and thallium) can further modify the scintillation properties of Cs_3_Cu_2_I_5_ [26, 27, 28]. For example, Mn‐doped Cs_3_Cu_2_I_5_ exhibited a several‐fold increase in light yield compared to the initial one because of multiple luminescence mechanisms, including STE, energy transfer, and impact excitation [26, 27]. In recent years, a growing number of promising metal halide scintillators have been reported to perform even better than commercial scintillators [13]. However, the extent to which these materials are close to real commercial applications still requires urgent validation.

In this study, we prepared Mn‐activated Cs_3_Cu_2_I_5_ powders via a simple solid‐state sintering route. Compared with the pristine material, a new emission band centered at 554 nm appears alongside the intrinsic STE luminescence. Combined with experimental evidence and density functional theory (DFT) calculations, we conclude that the new emission band arises from both direct excitation of Mn^2^^+^ ions and energy transfer from the STE state to Mn^2^^+^ ions. Besides, we directly evaluated the performance of Mn‐doped Cs_3_Cu_2_I_5_ with the CsI: Tl and DRZ‐Standard (DRZ‐std, i.e., Gd_2_O_2_S: Tb) commercial scintillators and demonstrated that Mn‐doped Cs_3_Cu_2_I_5_ possesses ideal light output, negligible afterglow, and a fast X‐ray excitation decay time of 46.4 µs. These characteristics enabled the X‐ray detectors integrated with Mn‐doped Cs_3_Cu_2_I_5_ to exhibit impressive detection performance. In particular, the spatial resolution of the copper halide TDI linear‐array detector reached 1.1 lp/mm (pixel size = 99 µm, 2 × 2 binning, magnification ratio: 1.02) in practical food and security inspections.

## 2 Results and Discussion

The Mn‐doped Cs_3_Cu_2_I_5_ polycrystalline powders were synthesized by a modified solid‐state reaction method [26, 29]. The Mn‐doped and undoped Cs_3_Cu_2_I_5_ discussed in this study are referred to hereafter as CCI: Mn and CCI, respectively. Briefly, CsI, CuI, and MnCl_2_·4H_2_O precursors were weighed in the desired molar ratios and thoroughly ground in an agate mortar to ensure homogeneous mixing. The mixture was then transferred to an alumina crucible and annealed at 370°C for 24 h under an inert gas atmosphere. The resulting powder was collected after the furnace was naturally cooled to room temperature. The crystal structure of CCI crystallizes in the orthorhombic Pnma space group and comprises two distinct Cu^+^ sites, namely the trigonal site and tetrahedral site, which together form [Cu_2_I_5_]^3^^−^ units [25, 30]. Each [Cu_2_I_5_]^3^^−^ cluster is spatially isolated by Cs^+^ ions, giving rise to a characteristic 0D electronic structure. As shown in Figure S1, the diffraction peaks of the CCI: Mn polycrystalline powders can be well indexed to the orthorhombic phase, indicating that the crystal structure remains unchanged upon Mn incorporation. Energy‐dispersive X‐ray spectroscopy confirms the homogeneous distribution of the constituent elements within the powders (Figure S2). The powder color gradually changes from off‐white to yellow with increasing Mn concentration (Figure S3). Under 365 nm UV excitation, the CCI: Mn powders exhibit bright green emission.

**FIGURE 1:**
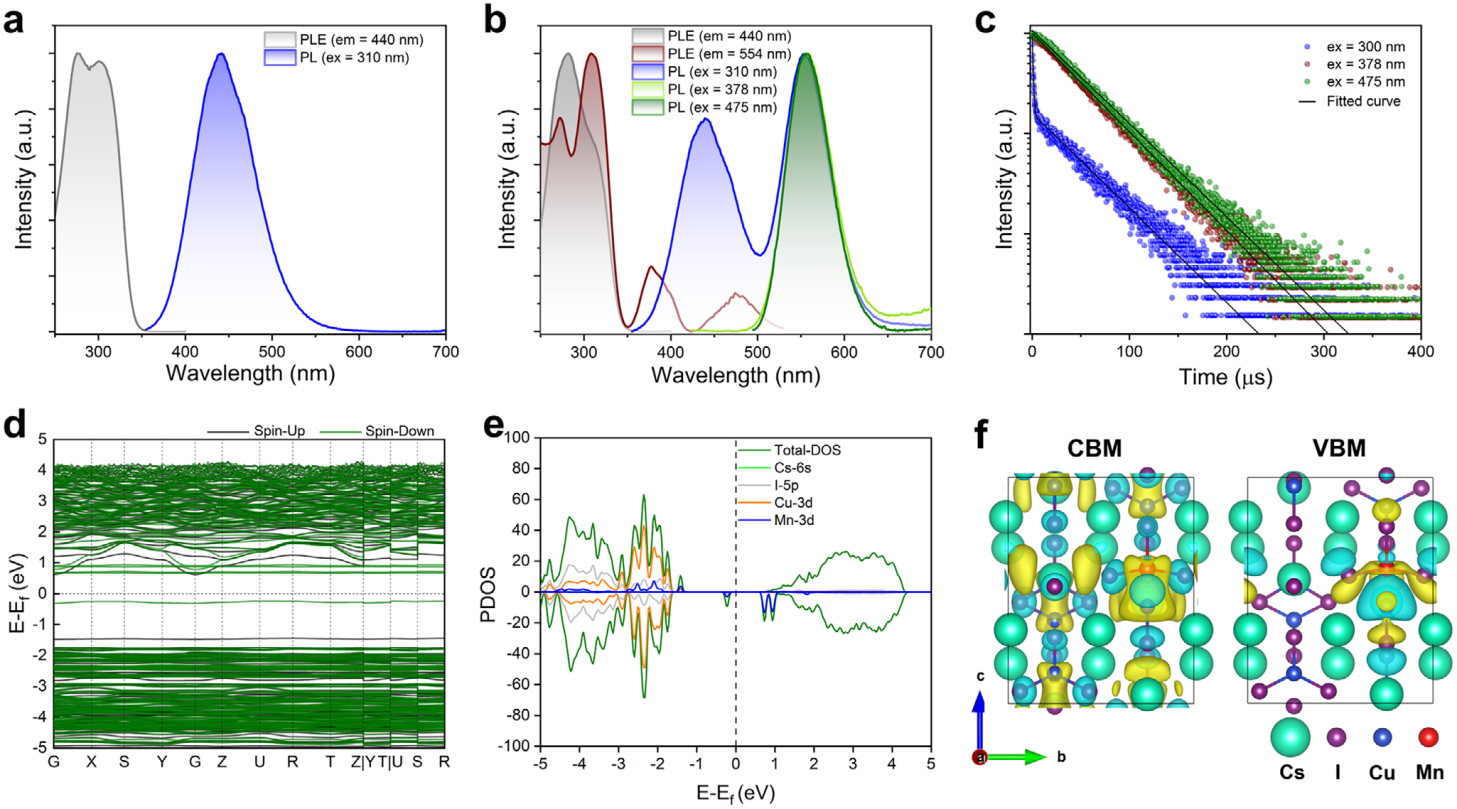
PL and PLE spectra of the (a) CCI and (b) CCI: Mn polycrystalline powders. (c) Time‐resolved PL decays of the CCI: Mn polycrystalline powders monitored at 554 nm. (d) Electronic band structure, (e) atoms projected density of states (PDOS), and (f) Electronic charge density for the CBM and VBM of CCI: Mn (Mn^2+^ ions occupy the Cu trigonal site).

Steady‐state photoluminescence (PL), PL excitation (PLE) spectra, and time‐resolved PL decays were recorded to investigate the optical properties of the synthesized CCI and CCI: Mn (7.5% molar ratio of Mn/Cs_3_Cu_2_I_5_) powders. As shown in Figure 1a, the CCI powders exhibit a PLE spectrum with a peak around 300 nm and a blue emission centered at 440 nm, with a full width at half maximum (FWHM) of 80 nm under 310 nm excitation. The broad emission band and large Stokes shift are consistent with prior reports on CCI and closely resemble those of other low‐dimensional copper halides (e.g., Rb_2_CuBr_3_, Rb_2_CuCl_3_, and K_2_CuBr_3_), which can be attributed to the STE mechanism [20, 21, 25, 31, 32]. Upon photoexcitation, strong electron–phonon coupling and Jahn–Teller lattice distortions in this soft lattice promote efficient exciton self‐trapping, dissipating much of the excitation energy through lattice relaxation. The subsequent relaxation from the excited state to the STE manifold gives rise to the large Stokes shift [1, 30]. By contrast, in addition to the intrinsic STE emission located at ∼440 nm, the CCI: Mn shows a new broad emission band ranging from 500 to 650 nm and peaking at ca. 554 nm, which is associated with the ^4^T_1_ to ^6^A_1_ transition of doped Mn^2+^ ions (Figure 1b) [33]. The PLE spectrum monitored at 440 nm closely resembles that of CCI, exhibiting only the intrinsic excitation band. When monitored at 554 nm, three noticeable excitation bands are observed at 310, 378, and 475 nm, corresponding to the intrinsic absorption, ^6^A_1_ to ^4^T_2_ (D) and ^6^A_1_ to ^4^T_1_ (G) transitions of Mn^2+^ ions, respectively [34]. The PL lifetime of the new emission band is on the order of several tens of microseconds under both excitation modes (Figure 1c; Table S1). Time‐resolved PL monitored at 554 nm yields monoexponential lifetimes of 44.08 and 46.68 µs for excitation at 378 and 475 nm, respectively. In contrast, the decay at 554 nm under 300 nm excitation is biexponential with a fast component of about 0.92 µs and a slower one of about 46.04 µs. Meanwhile, the STE‐related PL lifetime is significantly shortened after Mn doping, decreasing from 1.07 to 0.89 µs, as shown in Figure S4. These findings indicate that the new emission band arises not only from the direct excitation of Mn^2+^ ions but also from energy transfer from STE to Mn^2+^ ions. Upon deep‐ultraviolet excitation, the CCI host transfers energy to the dopant Mn^2+^ ions via STE states, effectively forming an energy ladder that culminates in Mn^2+^ emission [26, 27]. More insight into the luminescence properties of the CCI and CCI: Mn comes from DFT calculations. The computed band gap value of CCI is 2.46 eV, which is consistent with previous reports [35, 36]. The projected density of states and electronic charge density distributions show that the valence band maximum (VBM) and the conduction band minimum (CBM) are localized within the [Cu_2_I_5_]^3^^−^ units, originating predominantly from the I 5p and Cu 3d orbitals, while Cs^+^ ions do not contribute to the VBM and CBM (Figure S5). When Mn substitutes for Cu at either trigonal or tetrahedral sites, the dopant introduces in‐gap defect states and markedly narrows the band gap relative to the pristine CCI. A new Mn 3d‐derived electronic transition pathway emerges, giving rise to the Mn d‐d transition (Figure 1d–f; Figure S6) [36].

**FIGURE 2:**
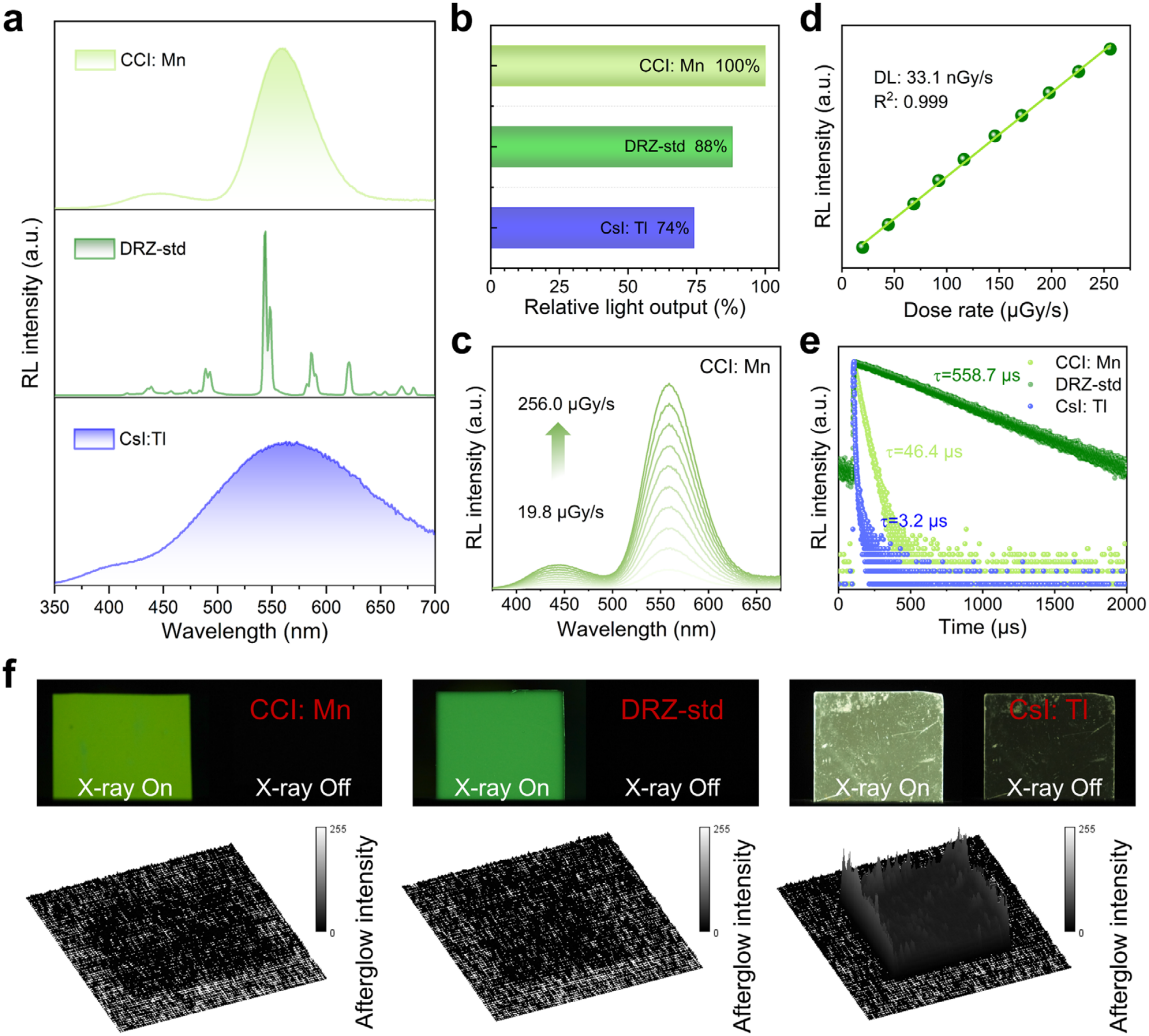
(a) RL spectra of the CCI: Mn film, commercial DRZ‐std scintillator, and CsI: Tl scintillation wafer. (b) Comparison of their integrated light output intensities based on the RL spectra. (c) Dose rate‐dependent RL spectra of the CCI: Mn film. (d) Detection limit of the CCI: Mn film. (e) RL decay spectra of the CCI: Mn, DRZ‐std, and CsI: Tl scintillators under X‐ray excitation. (f) Photographs of the scintillation light with X‐ray off (above) at consecutive 0.4 s exposures. Surface afterglow intensity maps (below) extracted from their X‐ray‐off photographs.

We then monitored the X‐ray‐induced radioluminescence (RL) spectra of quantitative CCI: Mn with different doping concentrations and determined the optimal concentration of 7.5% (Mn/Cs_3_Cu_2_I_5_ molar ratio) by comparing their integrated RL intensity (Figure S7). As the sample with 7.5% Mn doping exhibited excellent X‐ray resistance under a dose rate of 256.0 µGy s^−1^ as well as good thermal and humidity stability, we selected it as a representative of CCI: Mn for subsequent discussion (Figure S8). To verify the X‐ray imaging scintillation performance of the CCI: Mn halide, the CCI: Mn‐poly(methyl methacrylate) film, with a mass ratio of 70% CCI: Mn, was prepared through the blade‐coating method. As displayed in Figure 2a, we measured X‐ray‐induced RL spectra to compare the relative light output of the CCI: Mn film with that of scintillator references, including DRZ‐std and CsI: Tl. The CCI: Mn film exhibited bright green emission from Mn^2+^ ions under X‐ray excitation. The relative X‐ray‐induced emission intensity of the CCI: Mn film is 1.14 times greater than that of DRZ‐std and 1.35 times greater than that of CsI: Tl scintillators. The light yield of the CCI: Mn film is estimated to be approximately 72 900 photons MeV^−1^ as compared with the CsI: Tl scintillator (∼54000 photons MeV^−1^), demonstrating the high X‐ray to visible light conversion efficiency of the CCI: Mn halide (Figure 2b). Figure 2c shows that the dose‐dependent RL intensities of CCI: Mn film exhibit a well‐linear correlation with X‐ray dose rates from 19.8 to 256.0 µGy s^−1^. A detection limit of 33.1 nGy s^−1^ was achieved, which is more than two orders of magnitude lower than the typical dose rate used in standard medical diagnosis (5.5 µGy s^−1^) (Figure 2d).

In practical settings such as medical imaging and industrial non‐destructive testing, the detector time resolution and count rate are dictated primarily by the response time of the scintillator to excitation by high‐energy radiation or particles [37]. We then measured the RL decay time of CCI: Mn film under a pulsed X‐ray source (Figure 2e). The decay time of CCI: Mn film is 46.4 µs, much shorter than that of DRZ‐std (558.7 µs), while slightly longer than that of CsI: Tl (3.2 µs). Moreover, we judged their afterglow level by RL photographs with X‐ray on and off during a continuous 0.4 s exposure time. The RL of the CCI: Mn film has completely decayed to a dark background, while a distinct afterglow remains visible in CsI: Tl (Figure 2f). The afterglow level is a crucial parameter in X‐ray imaging applications, particularly for linear‐array detectors that perform multiple scans in rapid succession when monitoring moving objects. Insufficient attenuation of scintillator afterglow can result in image ghosting and distortion [38, 39]. These scintillation characteristics, including excellent light output and low detection limit (Table S2), as well as balanced RL decay and afterglow intensity, demonstrate that CCI: Mn is a promising candidate for high‐frame‐rate and fast X‐ray imaging applications.

**FIGURE 3:**
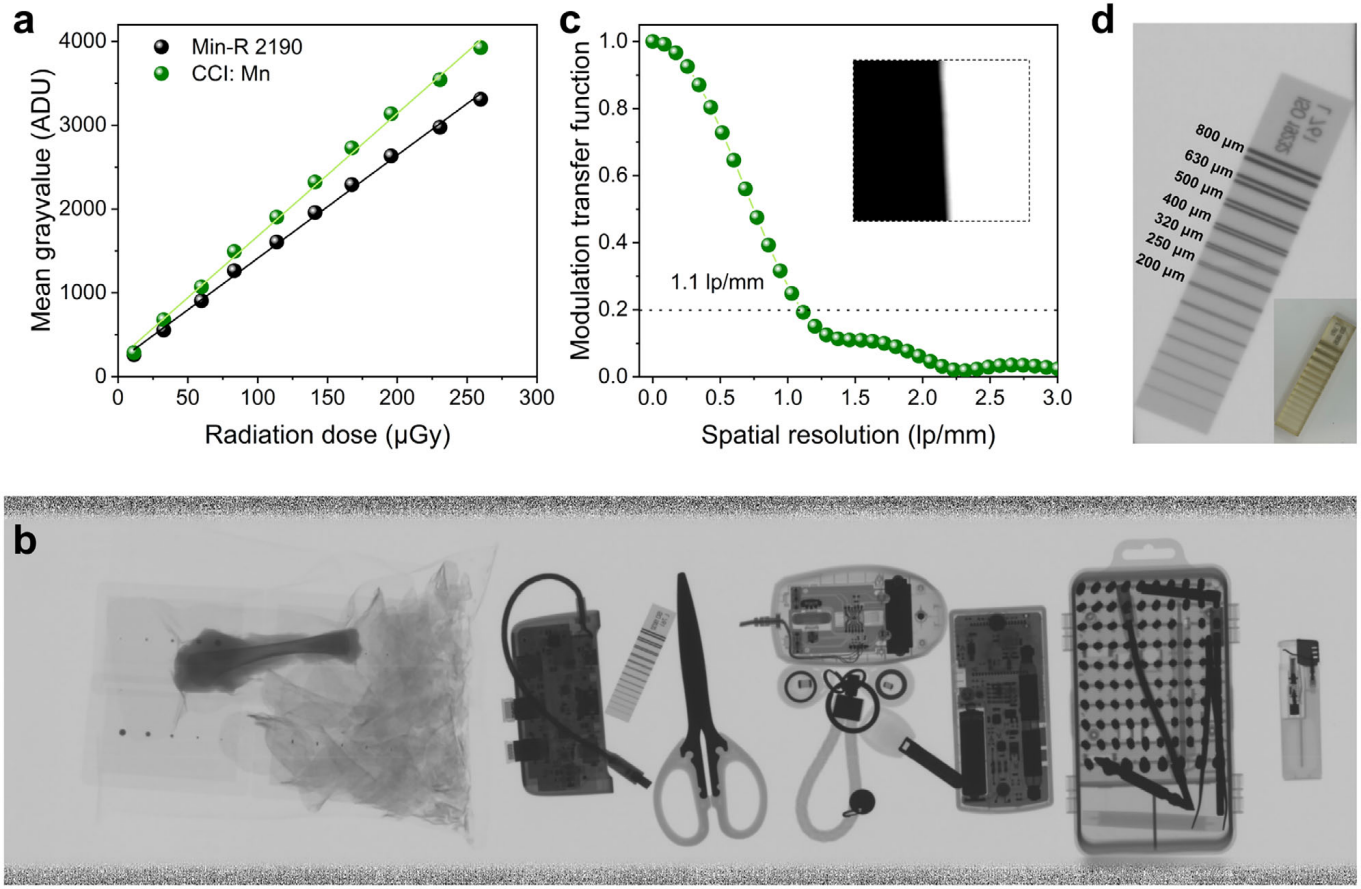
(a) Radiation dose‐dependent gray value mean of flat‐panel X‐ray detector with a commercial scintillator (Carestream Min‐R 2190) and CCI: Mn scintillator. (b) X‐ray image taken by the TDI linear‐array detector based on the CCI: Mn scintillator. (c) Modulation transfer function curve of the CCI: Mn scintillator with TDI linear‐array X‐ray detector measured by the slanted‐edge method. (d) Bright‐field and X‐ray image of the double‐wire image quality indicator.

We assembled the CCI: Mn film with a flat‐panel detector to evaluate the real‐life X‐ray detection capability. Interestingly, the light output of the CCI: Mn film is approximately 118% of that of a commercial imaging scintillator (Carestream Min‐R 2190) as estimated at the detector level, resulting in the detector being more sensitive across a broad range of X‐ray doses from 11.4 to 259.7 µGy (Figure 3a; Figure S9). We next demonstrated the X‐ray imaging capability of the flat‐panel detector with CCI: Mn film. A series of objects, such as a chip and a headphone, yielded clear internal structural information under X‐ray irradiation, as collected by the flat‐panel detector (Figure S10). The spatial resolution exceeds 3 lp/mm with a modulation transfer function (MTF) value of 0.2 (Figure S11). Recent progress has demonstrated that the lead‐free perovskite single‐crystal linear‐array detector can achieve high sensitivity and spatial resolution via direct conversion mode, highlighting great potential for high‐resolution imaging applications [40]. Nevertheless, indirect scintillator‐based linear‐array detectors remain highly attractive for practical deployment, as they offer superior operational stability, compatibility with mature readout electronics, and suitability for large‐area, high‐speed imaging. Motivated by these considerations, the CCI: Mn scintillator was therefore integrated with a commercial TDI linear‐array detector to demonstrate its applicability in food and security inspections. Then, the CCI: Mn scintillator‐based detector was placed beneath the conveyor belt, with the scanning frequency synchronized to the transport speed to ensure high imaging clarity and optimal sample throughput. A variety of objects, including food items and scissors, were used as test samples (Figure S12). These inspected objects moved along the conveyor belt, and their X‐ray images were continuously scanned as they passed through the TDI linear‐array detector. As a result, the captured images were sequentially stitched to generate a large‐scale linear‐array X‐ray image, as shown in Figure 3b. The achievable spatial resolution is governed by the intrinsic lateral optical crosstalk of the scintillator and characteristics of the imaging system. The measurements were performed using the TDI linear‐array detector with a native pixel pitch of 99 µm. To accommodate conveyor‐belt speeds in realistic inspection scenarios, 2 × 2 pixel binning was applied, resulting in an effective pixel pitch of approximately 200 µm. Without any optical magnification, the maximum achievable spatial resolution is therefore limited to 2.5 lp/mm. The spatial resolution was determined to be 1.1 lp/mm, based on an MTF value of 0.2 (Figure 3c). We can clearly distinguish foreign objects with a diameter of 0.3 mm in the food (Figure S13). Moreover, a spatial resolution better than 250 µm was clearly distinguished in a double‐wire image quality indicator (Figure 3d). These prototype detector‐level experiments for nondestructive food and security inspection demonstrate the significant potential of the CCI: Mn halide as a high‐performance imaging scintillator.

## 3 Conclusion

In conclusion, our findings indicate that CCI: Mn exhibits competitive X‐ray imaging performance compared to commercial scintillators such as CsI: Tl and DRZ‐Std. The higher light output significantly improves the performance of X‐ray detectors equipped with the CCI: Mn scintillator compared to the commercial Min‐R 2190 scintillator. Moreover, the negligible afterglow and rapid RL decay time of 46.4 µs equip the CCI: Mn scintillator for efficient rapid scanning X‐ray imaging. Notably, we successfully developed a CCI: Mn‐based linear‐array detector prototype that achieved a remarkable spatial resolution of 1.1 lp/mm in practical applications for food safety and security inspections, marking a significant advancement in the application field of novel metal halide scintillators.

## 4 Experimental Section/Methods

### 4.1 Materials

CuI (99.999%, Sigma–Aldrich), CsI (99.999%, Sigma–Aldrich), MnCl_2_·4H_2_O (99.5%, VWR Chemicals), poly(methyl methacrylate) (PMMA, MW∼40000, Macklin), UV‐curable adhesive (JINSHIDA, K‐6082), and ethyl acetate (EA, VWR Chemicals) were used without further purification. CsI: Tl and DRZ‐standard commercial scintillators were purchased from OST Photonics and Mitsubishi, respectively.

### 4.2 Synthesis of Mn‐Doped Cs_3_Cu_2_I_5_ Polycrystalline Powders

The Mn‐doped Cs_3_Cu_2_I_5_ (CCI: x%Mn, x% denotes the molar ratio of Mn to Cs_3_Cu_2_I_5_) polycrystalline powders were prepared via a solid‐state reaction method. CsI, CuI, and MnCl_2_·4H_2_O, weighted in the desired ratios in a glovebox, were well mixed in an agate mortar with a pestle and transferred into alumina crucibles. The mixtures were then annealed at 370°C for 24 h in a tubular furnace under an inert gas atmosphere. After the reaction was finished and naturally cooled to room temperature, the product powders were collected and stored in a glovebox for further use.

### 4.3 Fabrication of Films Based on the Mn‐Doped Cs_3_Cu_2_I_5_ Polycrystalline Powders

The Mn‐doped Cs_3_Cu_2_I_5_ polycrystalline powders were introduced into an ethyl acetate solution of PMMA, with a mass ratio of Mn‐doped Cs_3_Cu_2_I_5_ to PMMA of 7:3. A well‐mixed colloidal solution can be formed after thorough stirring. The films can be fabricated by blade‐coating the colloidal solution, followed by the ethyl acetate slowly evaporating at room temperature. For the films as scintillation layers combined with a linear‐array detector, the Mn‐doped Cs_3_Cu_2_I_5_ polycrystalline powders and curable adhesive were mixed in an agate mortar. Then, the colloidal mixture uniformly adhered to the PET substrate through the blade‐coating method, followed by UV curing to form the films. The mass ratio of the powders to the curable adhesive is 7:3.

### 4.4 Characterization

X‐ray diffraction (XRD) patterns were recorded at room temperature using a Bruker D8 ADVANCE diffractometer with Cu Kα radiation (λ = 1.5406 Å). Scanning electron microscopy (Zeiss Auriga) with an energy‐dispersive X‐ray analyzer was used to analyze the chemical compositions of powder samples. The photoluminescence (PL) and PL excitation (PLE) spectra were taken using a Horiba Fluoromax‐4 spectrofluorometer with a photomultiplier (PMT‐928). Time‐resolved PL measurements monitored at 440 nm were acquired by the time‐correlated single‐photon counting (TCSPC) technique in a Halcyone setup (Ultrafast Systems). The samples were excited with a 300 nm pulsed laser beam coming from an optical parametric amplifier (Newport Spectra‐Physics) seeded with an Astrella femtosecond pulsed laser (150 fs, 800 nm 1 kHz, Coherent). Time‐resolved PL measurements monitored at 554 nm were performed on the FS5‐Edinburgh. Samples were excited using a pulsed microsecond flash lamp. The lifetime data were acquired using the Multi‐Channel Scaling (MCS) technique, and the decay curves were fitted to an exponential function to determine the lifetime. The X‐ray excited decay curve was measured by FS5‐Edinburgh, equipped with a pulsed X‐ray source. A pulsed X‐ray source was triggered by an HPL/VPL 450 nm laser. The pulsed X‐ray was set to 40 kV, 0.4 µA. For the afterglow measurement, the sample was initially excited by X‐rays at 50 kV and 80 µA for a duration of 10 s. Immediately after turning off the X‐ray source, the camera (Sony α7) began capturing images. Five consecutive photos were taken over 2 s to observe the sample's afterglow behavior.

### 4.5 Radioluminescence (RL) Studies

The RL spectra were measured and corrected by a spectrometer (Horiba Fluromax‐4) equipped with an X‐ray tube (Tungsten target, Moxtek). The detection slit was set at 5 nm, and the X‐ray outlet was set to 1 cm away from the sample for all spectral measurements. All tests were carried out in a radiation‐tight environment of lead‐plate shielding. The relative X‐ray‐induced emission intensity of Mn‐doped Cs_3_Cu_2_I_5_ and CsI: Tl and DRZ‐standard commercial scintillators was evaluated by comparing the integrated area of their RL spectra under the X‐ray excitation with the same dose rate.

### 4.6 Calculation of X‐Ray Detection Limit

The linear relationship between the RL intensity of the corresponding samples and the X‐ray dose rate was obtained. The noise data was measured in the absence of a sample. The noise intensity value was statistically analyzed and fitted by the Gaussian function, whereby the FWHM was regarded as the average noise value. The detection limit in dose rate was derived from the slope of the fitting line, with a signal‐to‐noise ratio of 3.

### 4.7 X‐Ray Image Collection and Process

A flat‐panel detector (Remote RadEye HR) and a TDI linear‐array detector (Shad‐o‐scan 3001) were employed for X‐ray imaging using scintillation layers based on Mn‐doped Cs_3_Cu_2_I_5_. For the flat‐panel detector measurements, the X‐ray source was operated at 70 KV and 10 µA with an exposure time of 1 s, and the greyscale output ranged from 0 to 4096 (12 bit). Linear‐array detector measurements were conducted on a prototype food inspection system with a focus object distance of 460 mm and a focus detector distance of 490 mm. A Shad‐o‐scan scanning X‐ray detector with a 99 µm pixel pitch was positioned beneath the conveyor belt of the industrial inspection setup. The scintillator layer was optically coupled to the complementary metal‐oxide‐semiconductor (CMOS) readout module using silicon grease. To minimize image distortion, the detector scanning frequency and frame rate were precisely synchronized with the conveyor belt speed. A circular metal object was first scanned to calibrate the system, confirming a belt speed of 10 meters per minute, after which 2 × 2 pixel binning was applied for optimal sample throughput and image clarity. The X‐ray source was operated at 60 kV and 2000 µA to ensure sufficient X‐ray penetration and contrast for accurate detection of sample features. The acquired X‐ray image taken by the linear‐array detector was further processed with flat‐field correction and rotation. All measurements were conducted in a radiation‐shielded environment.

### 4.8 Calculation of X‐Ray Imaging Spatial Resolution

X‐ray imaging spatial resolution was calculated using modulation transfer function (MTF) measurements. The MTF was estimated using the slanted‐edge method. MTF analysis was performed on the images via *Image J* software. The edge spread function (ESF) was derived from the edge image, and the line spread function (LSF) was derived from the derivation. Finally, the Fourier transform of LSF defines MTF. The summary is shown in the following formula:

$$MTF\left(v\right)=F\left(LSF\left(x\right)\right)=F\left(\frac{dESF\left(x\right)}{dx}\right)$$

where *v* is the spatial frequency, and *x* is the position of pixels.

### 4.9 Density Functional Theory (DFT) Calculations

All DFT calculations were carried out with the Vienna Ab initio Simulation Package (VASP). The DFT calculations were treated within the generalized‐gradient approximation (GGA) using the Perdew–Burke–Ernzerhof (PBE) functional. Ionic cores were represented by projected augmented wave (PAW) potentials, and the valence electrons were treated using a plane‐wave basis with a kinetic‐energy cutoff of 450 eV. Partial occupancies of the Kohn–Sham orbitals were handled by Gaussian smearing with a width of 0.05 eV. For geometry optimization, Brillouin‐zone integrations employed a *Γ*‐centered *k*‐point sampling with 0.04/Å, and a 0.02/Å *Γ*‐centered *k*‐point sampling was used for density of states calculations. A convergence threshold of 10^−^^6^ eV was applied to the self‐consistent field calculations. Equilibrium geometries and lattice constants were optimized until the maximum stress exerted on each atom was below 0.02 eV Å^−^^1^. The weak interaction was included via the DFT‐D3 scheme of Grimme. Spin polarization was enabled to describe magnetic systems.

## Author Contributions

Yang Zhou and Omar F. Mohammed conceived the idea. Yang Zhou, Tengyue He, and Wenyi Shao prepared materials, performed the scintillation measurements, and analyzed the data. Wenyi Shao performed and analyzed the X‐ray imaging measurements. Wentao Wu and Peng Yuan helped with photophysical characterization. Haijiao Xie carried out DFT calculations. Osman M. Bakr contributed to the discussion of the experimental data. Yang Zhou wrote the manuscript. Omar F. Mohammed supervised this project. All authors discussed the results and contributed to manuscript revisions.

## Conflicts of Interest

The authors declare no conflicts of interest.

## Supporting information




**Supporting File**: smll72740‐sup‐0001‐SuppMat.docx.

## Data Availability

The data that support the findings of this study are available in the supplementary material of this article.
