# Supplementary material for: Exploration of Copper Halide Linear‐Array Detector Prototype for Security Checks
Source: Small. 2026 Feb 5;22(20):e14814. doi: 10.1002/smll.202514814 (PMC13054205; doi:10.1002/smll.202514814)
Supplement: Supplementary file 1 — Supporting File: smll72740‐sup‐0001‐SuppMat.docx. [file SMLL-22-e14814-s001.docx]

Supporting Information

**Exploration of Copper Halide Linear-Array Detector Prototype for Security Checks**

Yang Zhou, Tengyue He, Wenyi Shao, Wentao Wu, Peng Yuan, Haijiao Xie, Osman M. Bakr, and Omar F. Mohammed*

Y. Zhou, T. He, W. Shao, W. Wu, P. Yuan, O. M. Bakr, O. F. Mohammed

Center for Renewable Energy and Storage Technologies (CREST), Division of Physical Science and Engineering, King Abdullah University of Science and Technology, Thuwal 23955-6900, Kingdom of Saudi Arabia

E-mail: [omar.abdelsaboor@kaust.edu.sa](mailto:omar.abdelsaboor@kaust.edu.sa) (Prof. O. F. Mohammed)

Y. Zhou

School of Materials Science and Engineering, University of Jinan, Jinan, 250022 Shandong, China

H. Xie

Hangzhou Yanqu Information Technology Co., Ltd., Xixi Legu Creative Pioneering Park, Hangzhou, 310003, Zhejiang, China


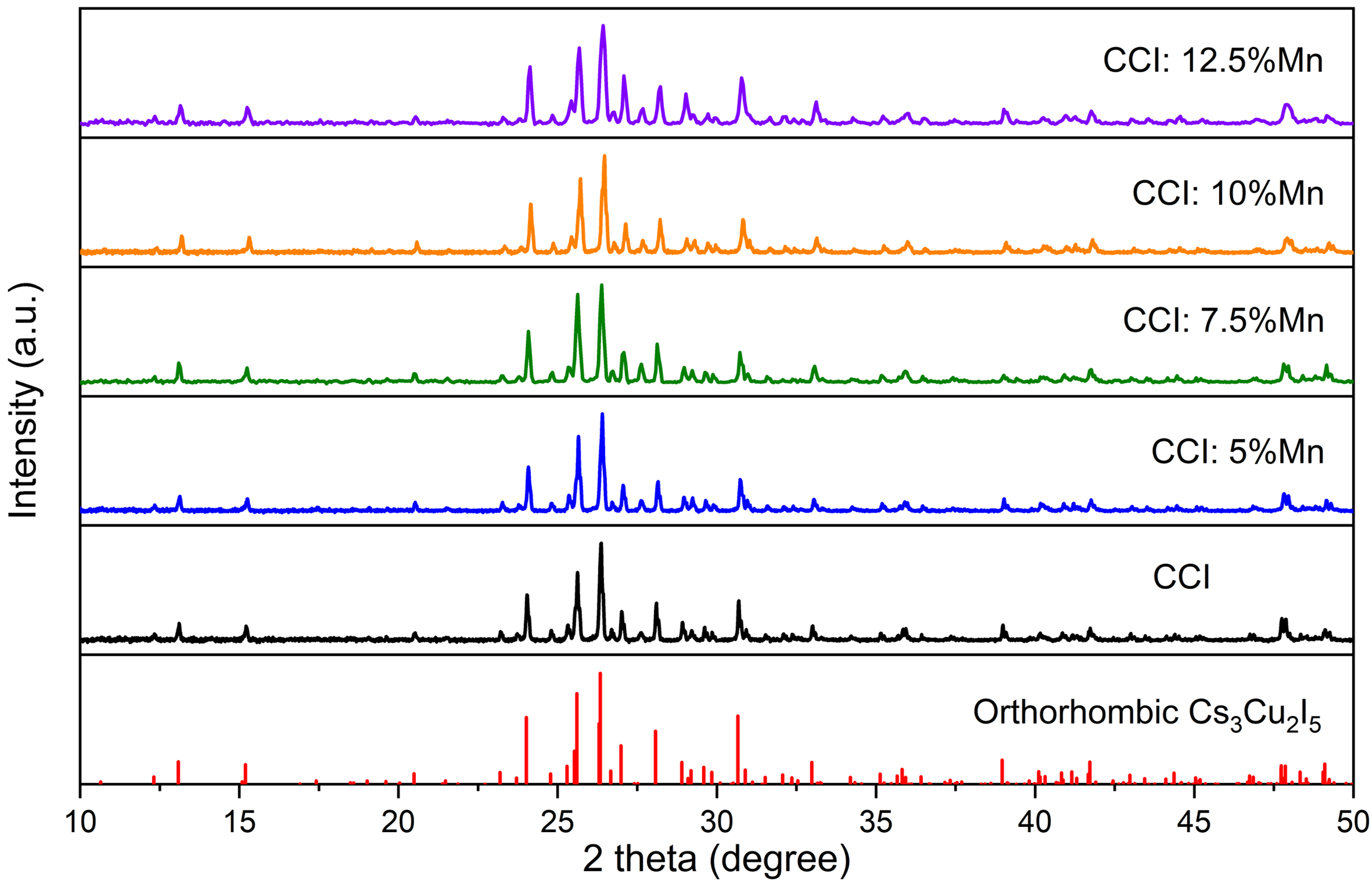


**Figure S1.** XRD patterns of the CCI: x%Mn polycrystalline powders.


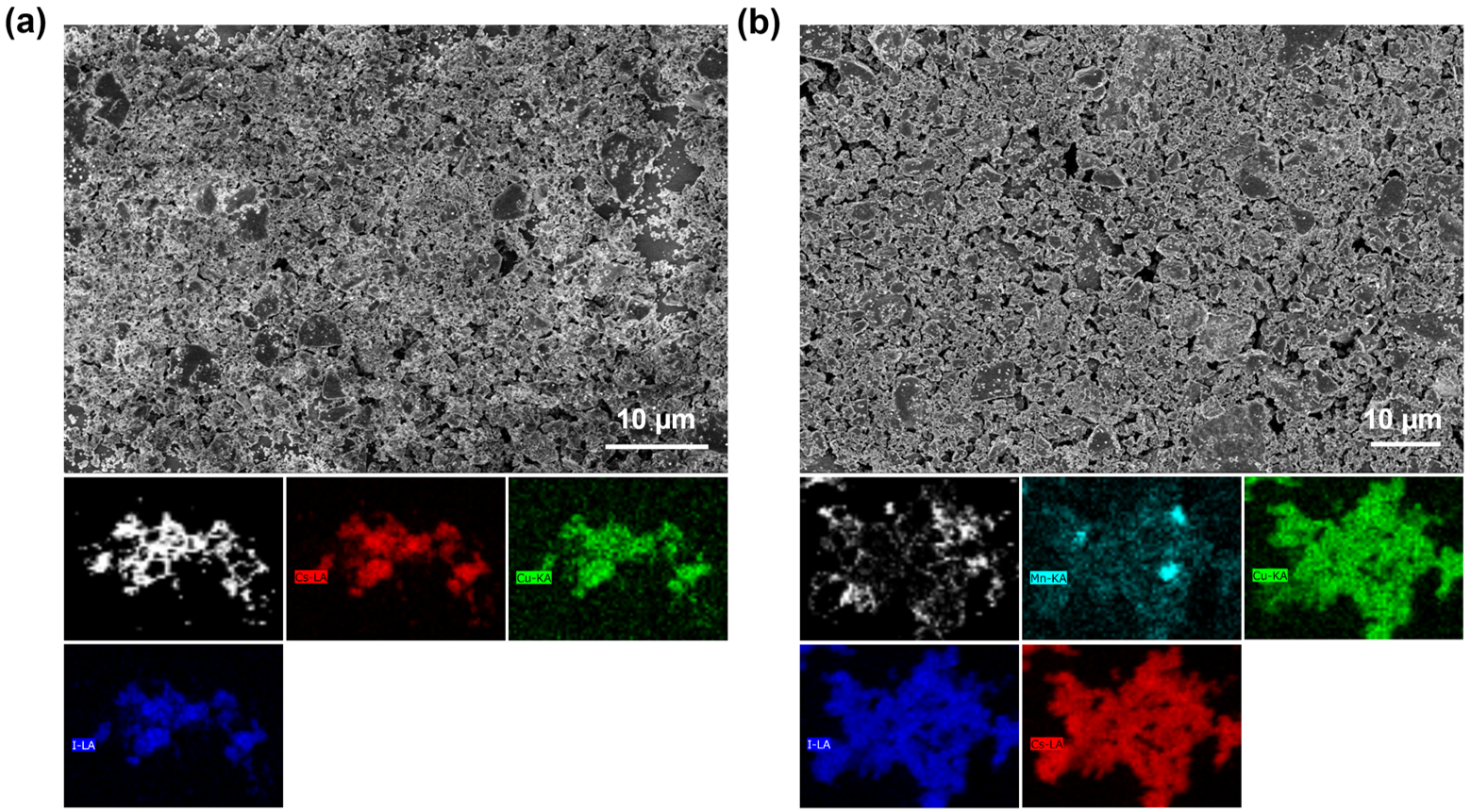


**Figure S2.** Scanning electron microscopy image and Energy-dispersive X-ray spectroscopy elemental mapping of the CCI (a) and CCI: Mn (b) ground powders.


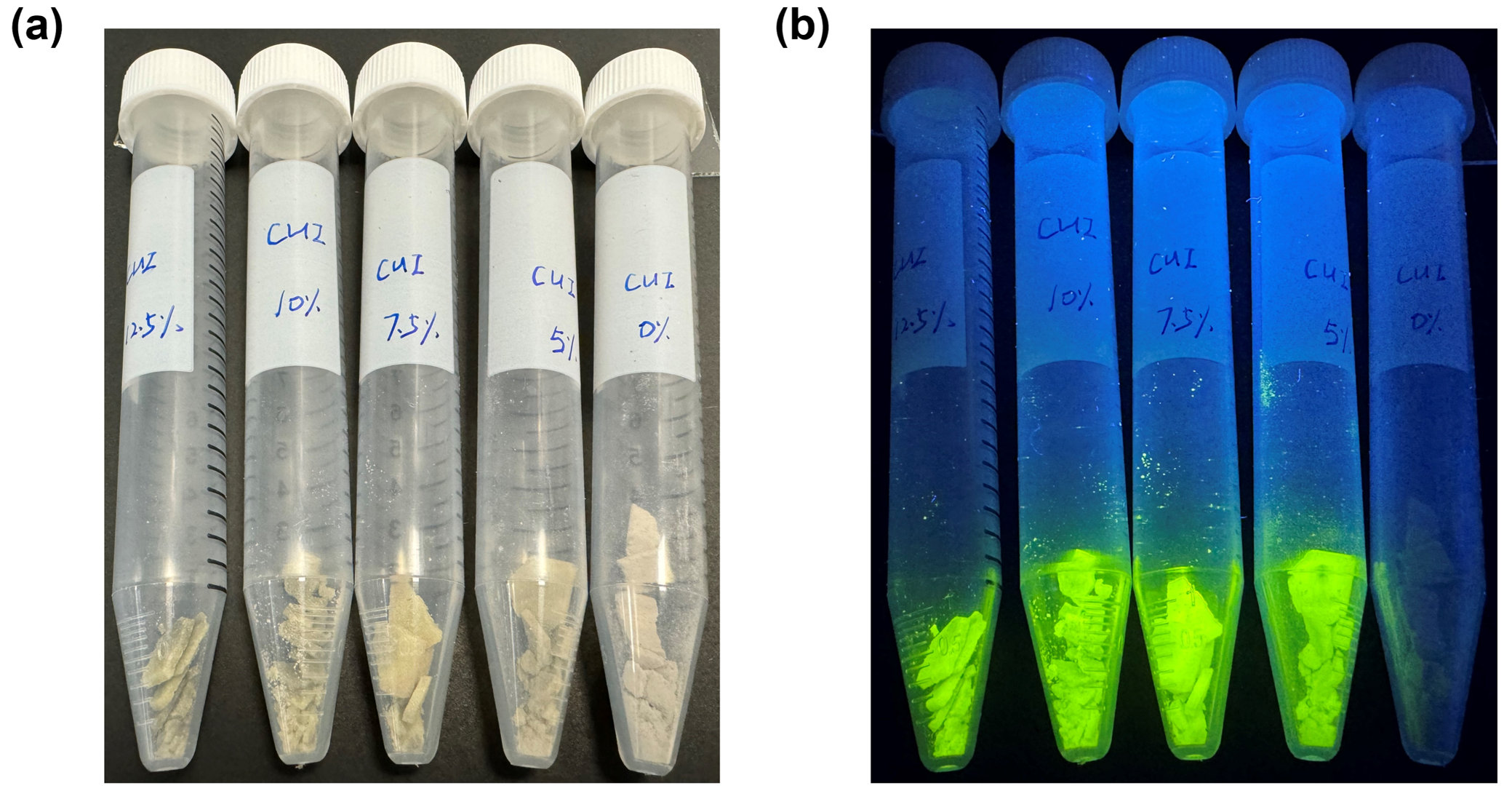


**Figure S3.** Photographs of the CCI: x%Mn polycrystalline powders under ambient (a) and 365 nm UV excitation (b).


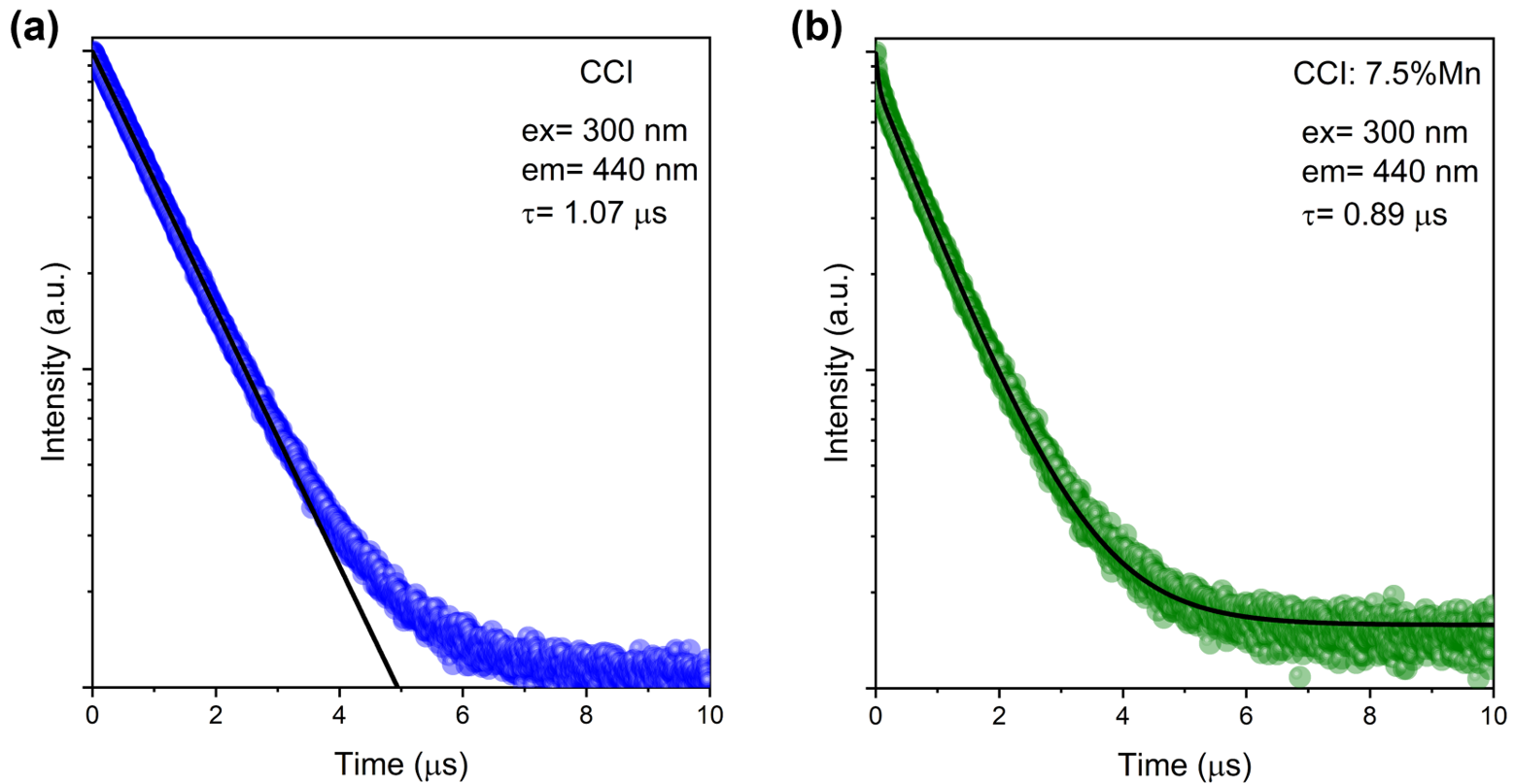


**Figure S4.** Time-resolved PL decays of the CCI (a) and CCI: 7.5%Mn (b) polycrystalline powders excited/monitored at 300 and 440 nm.


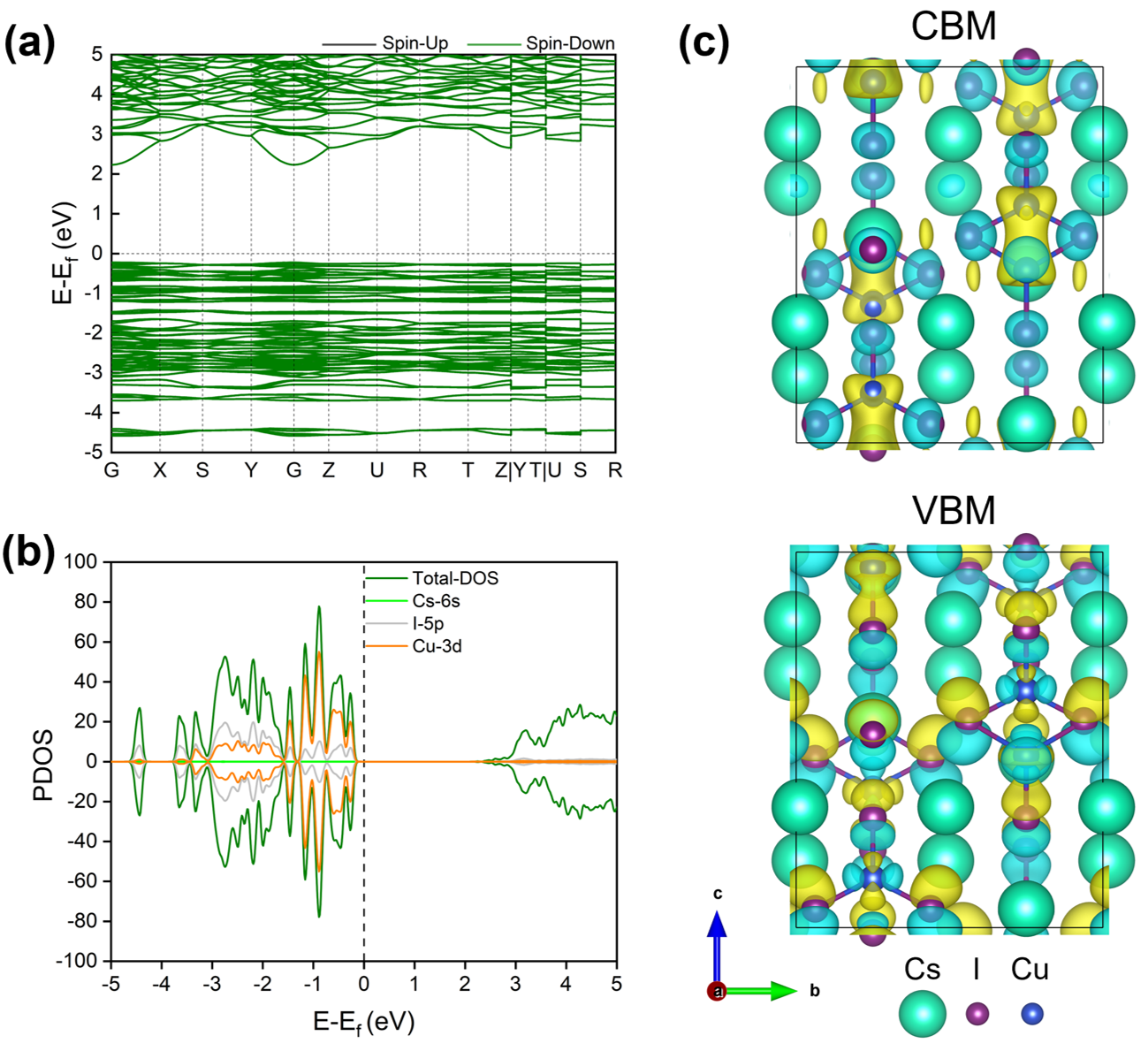


**Figure S5.** (a) Electronic band structure, (b) atoms projected density of states (PDOS), and (c) Electronic charge density for the conduction band minimum (CBM) and valence band maximum (VBM) of CCI.


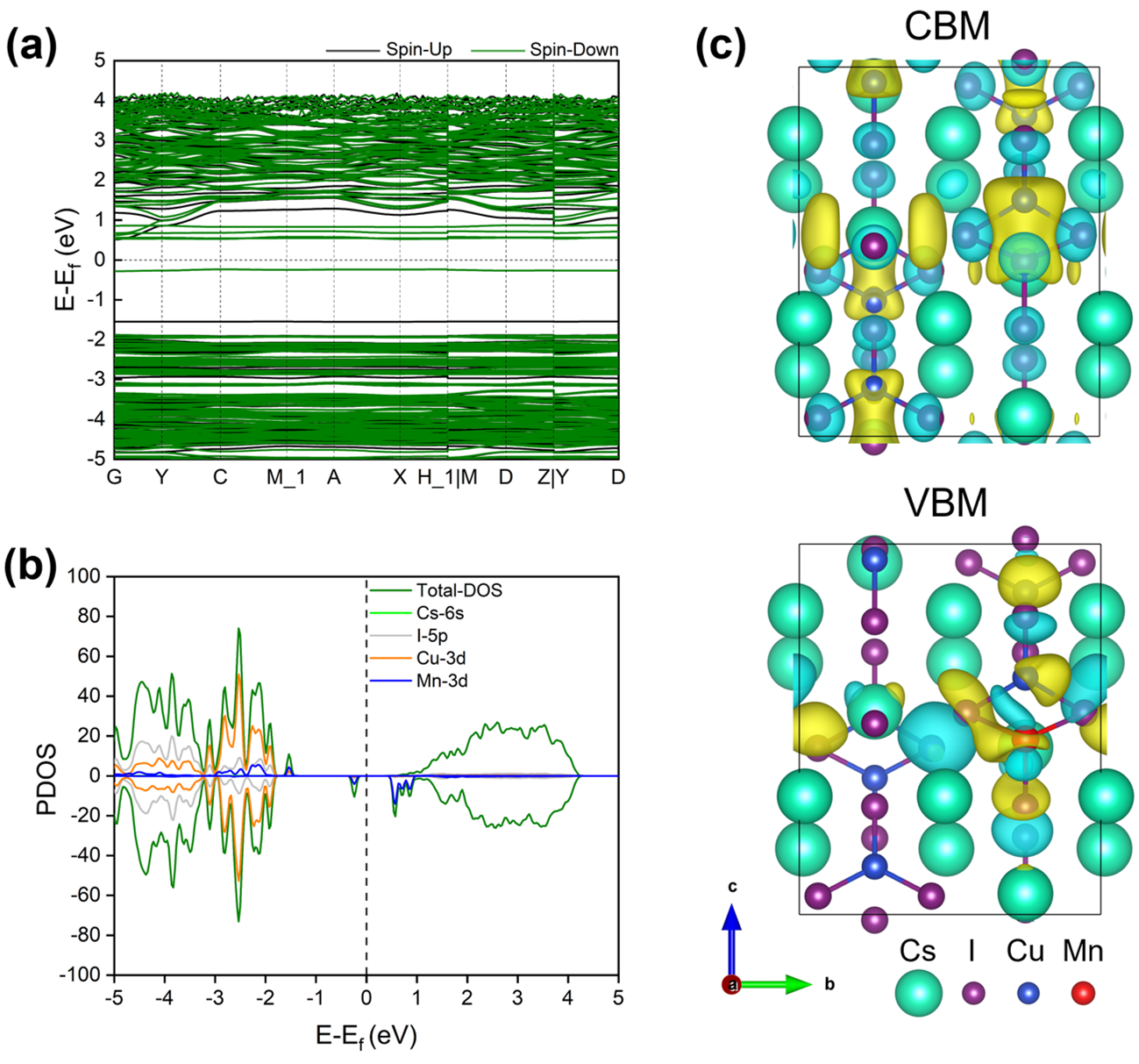


**Figure S6.** (a) Electronic band structure, (b) atoms projected density of states (PDOS), and (c) Electronic charge density for the conduction band minimum (CBM) and valence band maximum (VBM) of CCI: Mn (Mn^2+^ ions occupy the Cu tetrahedral site).


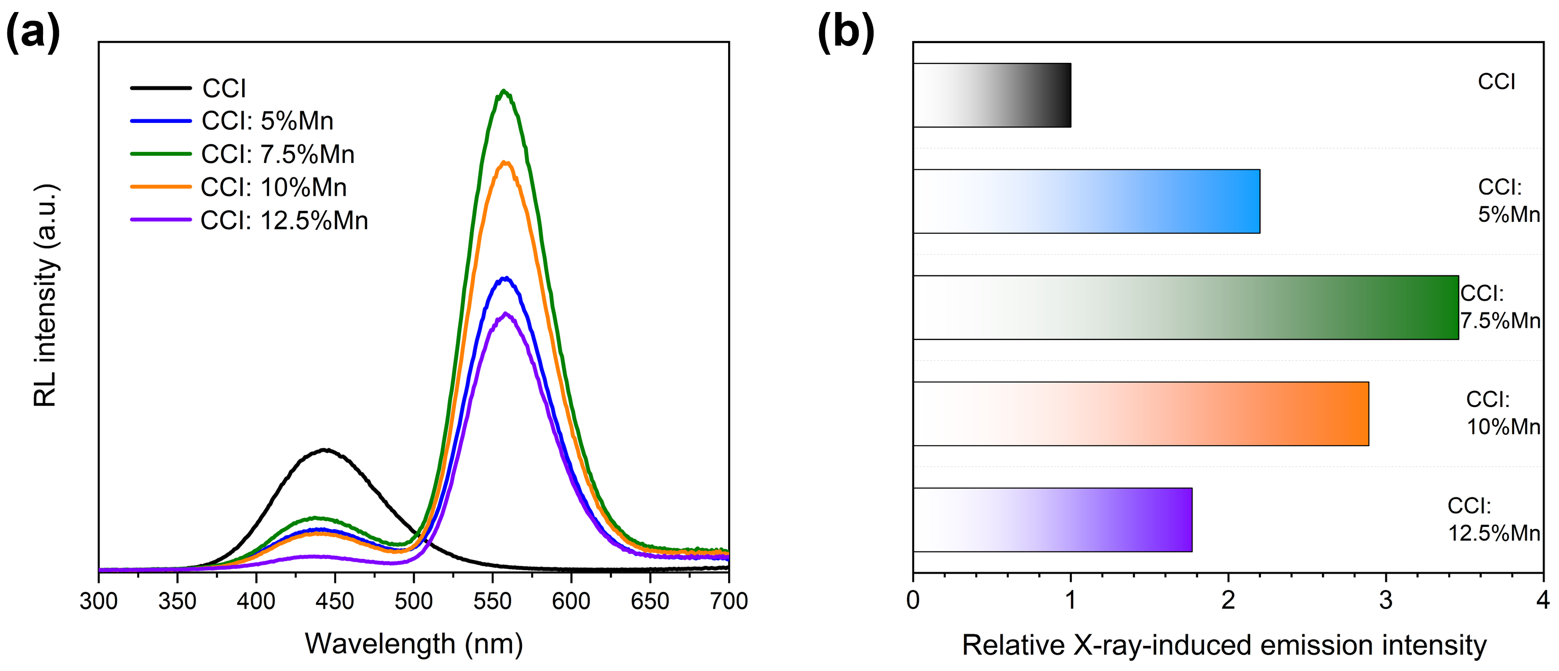


**Figure S7.** RL spectra (a) and relative X-ray-induced emission intensity (b) of the CCI: x%Mn polycrystalline powders.


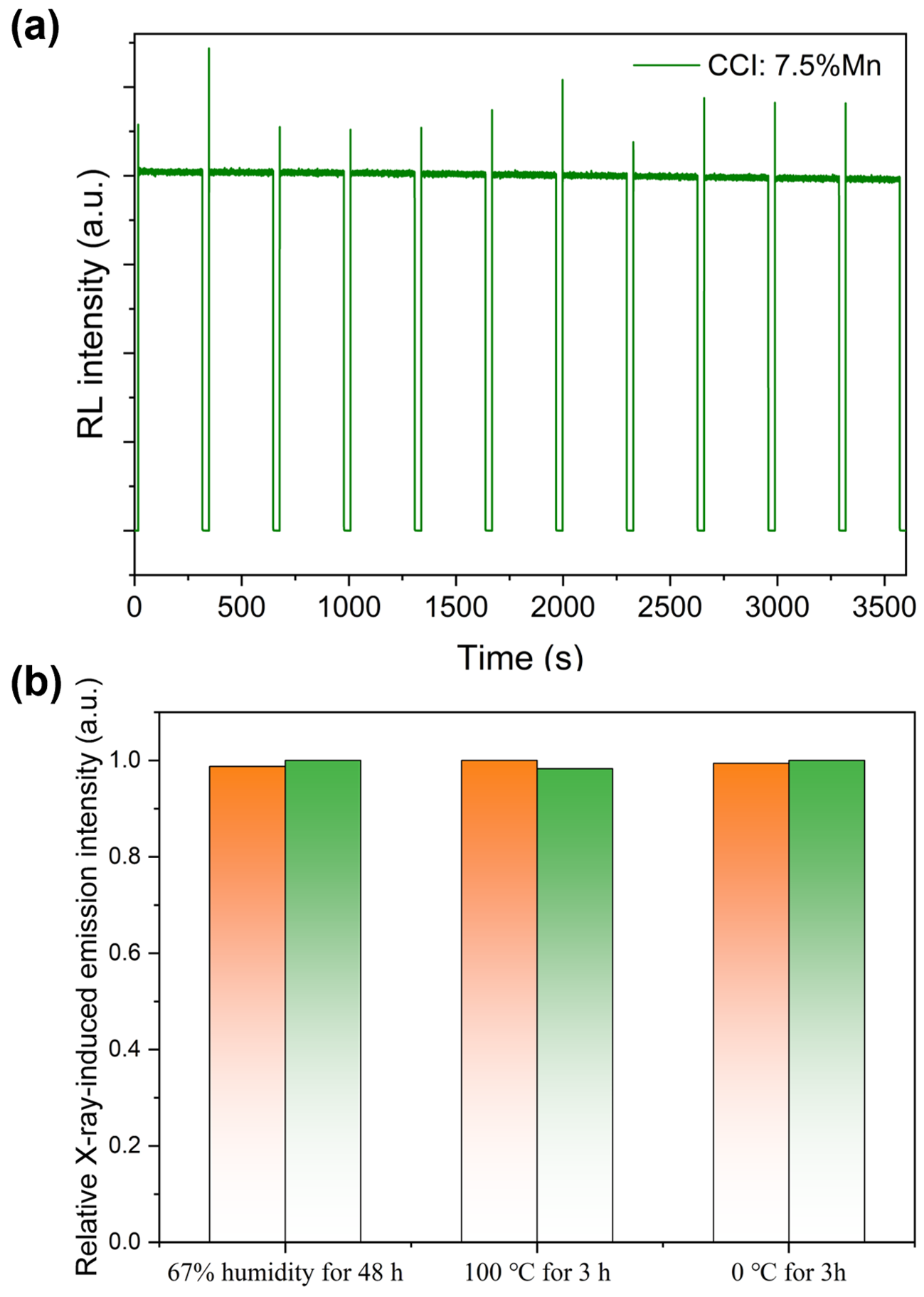


**Figure S8.** (a) Irradiation stability of the CCI: 7.5%Mn polycrystalline-based film under X-ray irradiation with a dose rate of 256.0 μGy s^−1^. (b) Changes in the relative X-ray-induced emission intensity of CCI: 7.5%Mn polycrystalline-based film at high-humidity (67% for 48 h) as well as high- and low-temperature (100 or 0 ℃ for 3 h) conditions.


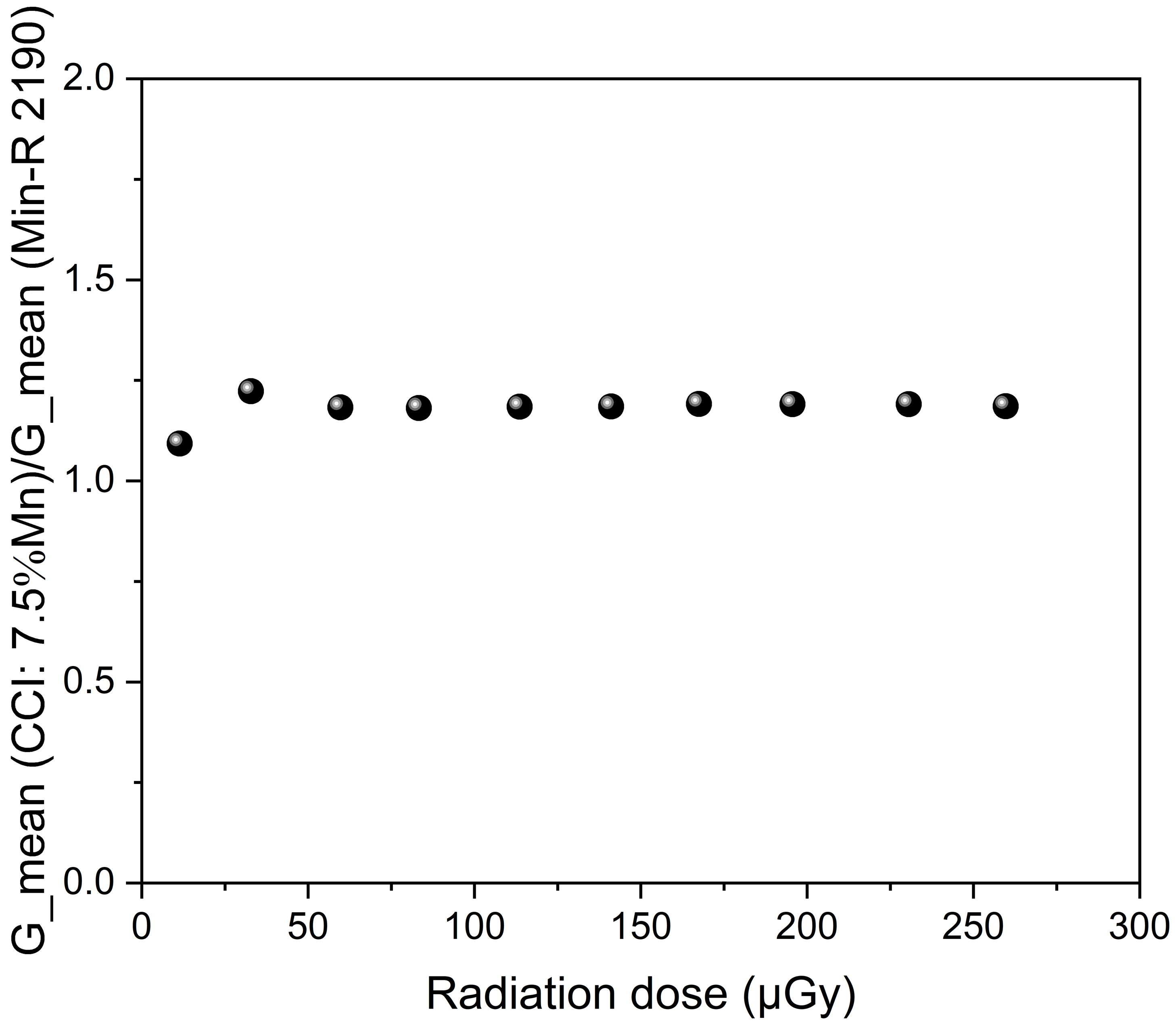


**Figure S9.** The ratio of the gray value mean (G_mean) of the flat-panel X-ray detector with CCI: Mn to that of the detector with Min-R 2190 scintillator.


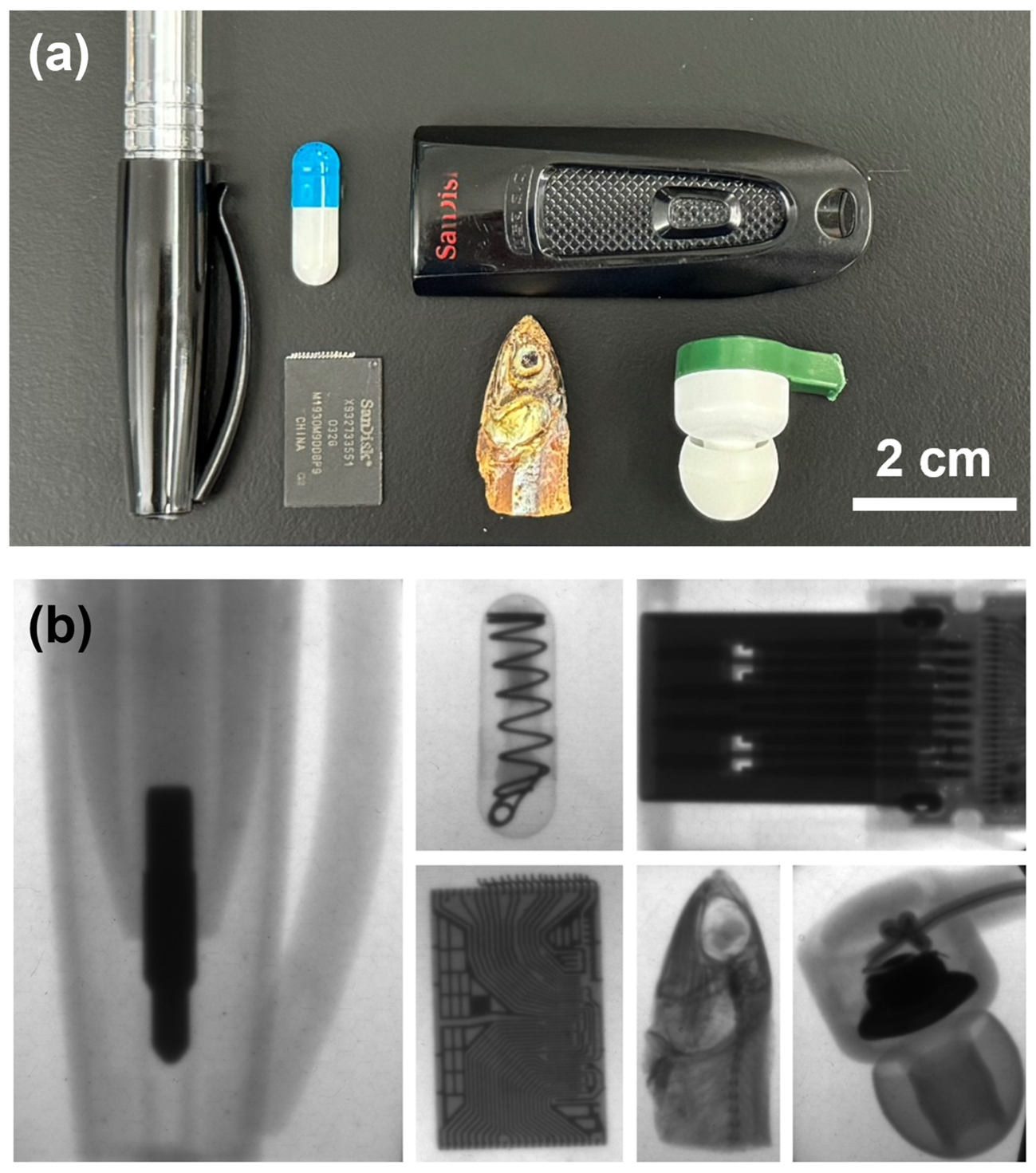


**Figure S10.** X-ray images of the objects taken by CCI: Mn scintillator based on a flat-panel detector (Remote RadEye HR).


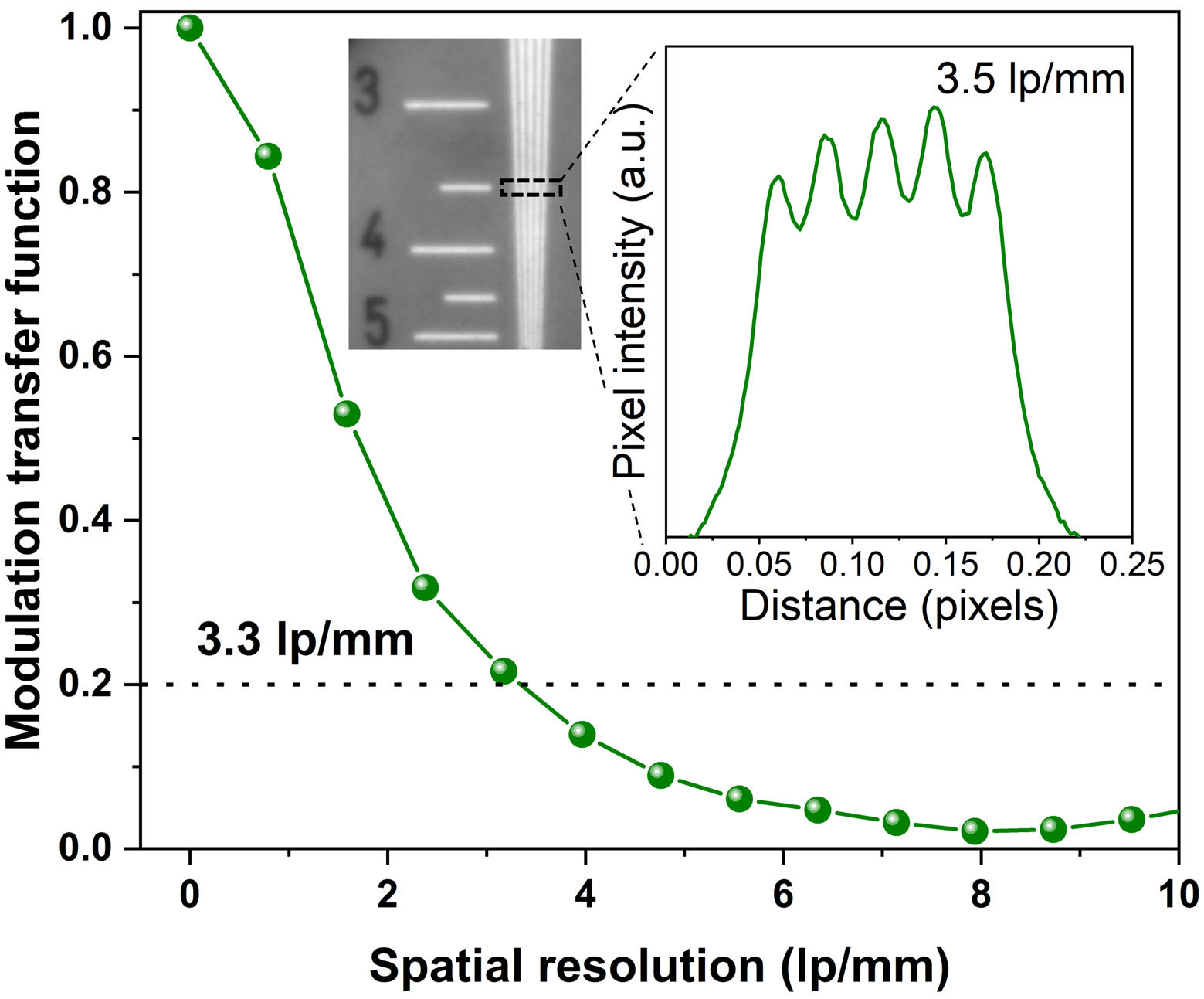


**Figure S11.** Modulation transfer function curve of the CCI: Mn scintillator based on a flat-panel detector measured by the slanted-edge method. The inset shows the gray value profiles of the line pair extracted from the line pair card.


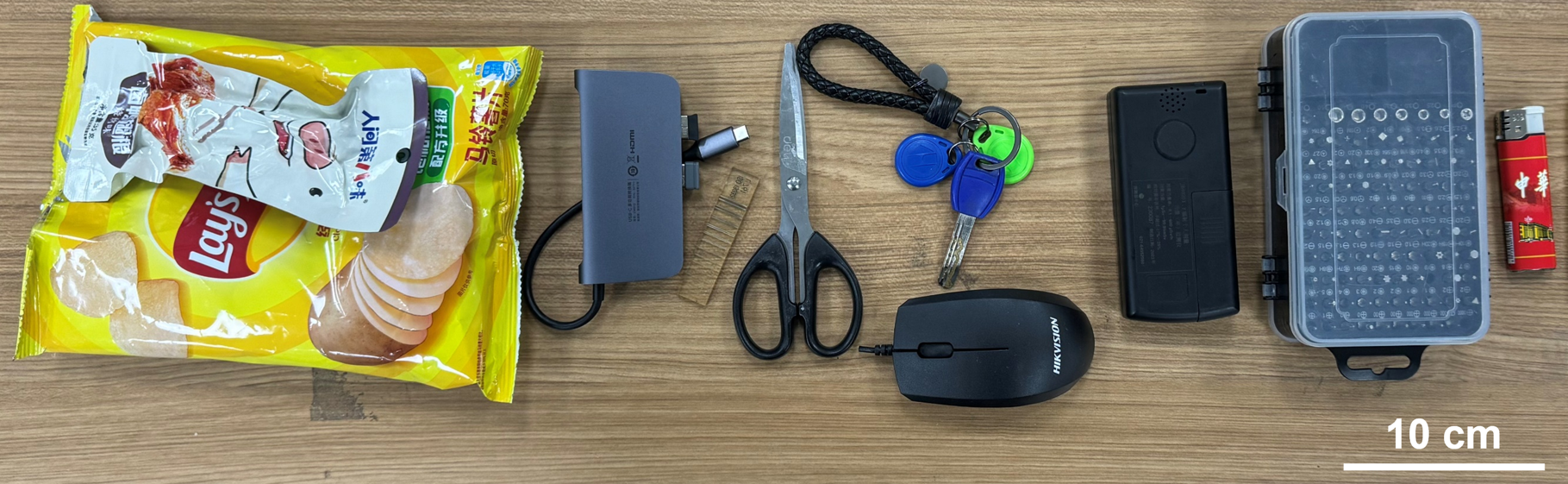


**Figure S12.** Photographs of the objects used for X-ray imaging with CCI: Mn scintillator based on TDI linear-array X-ray detector.


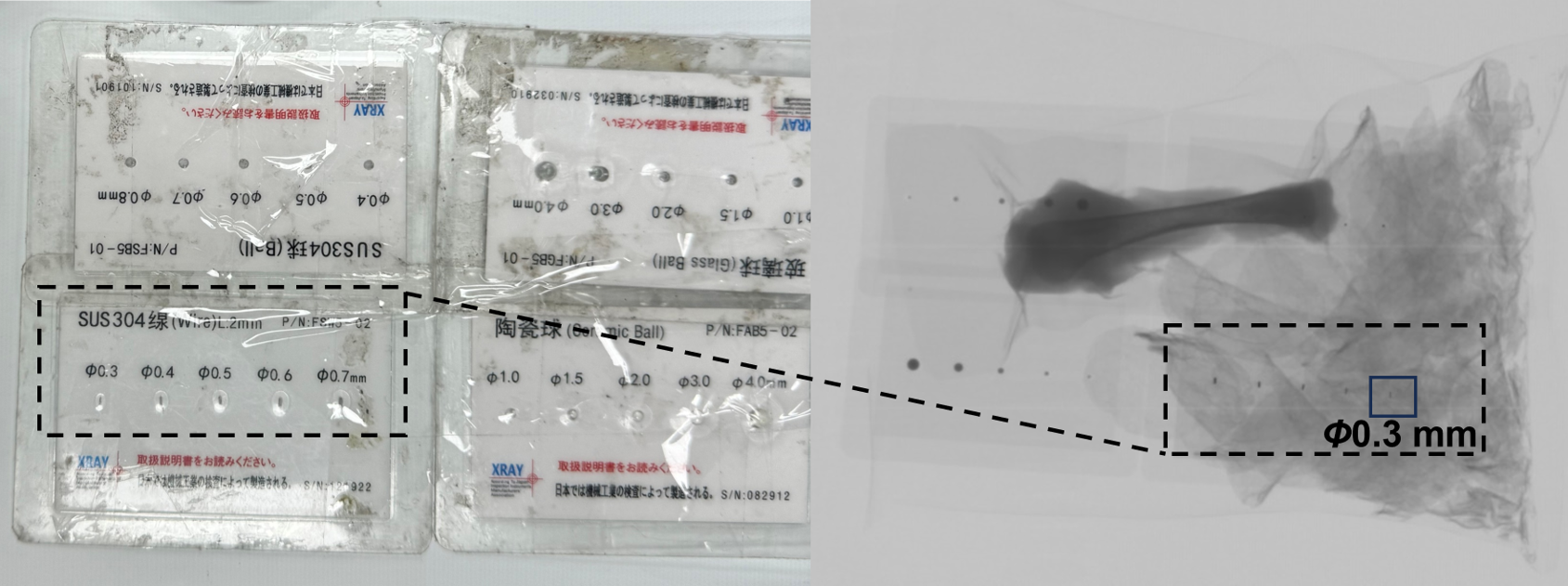


**Figure S13.** Bright-field and X-ray images of the foreign body standard card. Note that the X-ray image is extracted from Figure 3b.

**Table S1.** The fitted lifetimes of CCI: 7.5%Mn polycrystalline powders monitored at 554 nm by a mono- or multi-exponential function.

| Excitation wavelength  (nm) | A_1_ | t_1_ (µs) | A_2_ | t_2_ (µs) |
| --- | --- | --- | --- | --- |
| 300 | 0.783 | 0.916 | 0.159 | 46.040 |
| 378 | 1.008 | 44.077 |  |  |
| 475 | 1.072 | 46.675 |  |  |

**Table S2.** Comparison with the scintillation properties of several previously reported classic all-inorganic metal halide scintillators.

| Materials | Maximum emission (nm) | Light yield  (Photons MeV^−1^) | Detection limit  (nGy s^−1^) | ref |
| --- | --- | --- | --- | --- |
| CsPbBr_3_ colloid | 520 | ~21000 | / | 1 |
| Cs_3_Cu_2_I_5_ powder-based film | 445 | ~48800 | 48.6 | 2 |
| CsCu_2_I_3_ single crystals | 556 | 21580 | / | 3 |
| Cs_5_Cu_3_Cl_6_I_2_ film | 466 | 64800-67200 | 11 | 4 |
| Tl-doped Cs_3_Cu_2_I_5_-polymer film | ~510 | ∼48800 | 305 | 5 |
| Rb_2_AgBr_3_ single crystals | 480 | 25600 | 19 | 6 |
| Cu-doped Cs_2_AgI_3_-polymer film | ~470 | 55000 | 101.3 | 7 |
| Cs_3_MnI_5_ polycrystalline powders | 540 | 33600 | 400 | 8 |
| Cs_3_TbCl_6_ polycrystals | 548 | 56800 | 149.65 | 9 |
| Rb_3_TbCl_6_ polycrystals | 548 | 88800 | 115.38 |  |
| Cs_2_NaTbCl_6_ crystals | 548 | 46600 | / | 10 |
| Cs_2_NaEuCl_6_ crystals | 593 | 1250 | / |  |
| CCI: Mn-polymer film | ~558 | ∼72900 | 33.1 | **This work** |

**References**

1. Y. Zhang, R. Sun, X. Ou, K. Fu, Q. Chen, Y. Ding, L. Xu, L. Liu, Y. Han, A. V. Malko, X. Liu, H. Yang, O. M. Bakr, H. Liu, and O. F. Mohammed, *ACS Nano* **2019**, *13*, 2520−2525.
2. Y. Zhou, X. Wang, T. He, H. Yang, C. Yang, B. Shao, L. Gutiérrez-Arzaluz, O. M. Bakr, Y. Zhang, and O. F. Mohammed, *ACS Energy Lett.* **2022**, *7*, 844−846.
3. M. Zhang, J. Zhu, B. Yang, G. Niu, H. Wu, X. Zhao, L. Yin, T. Jin, X. Liang, and J. Tang, *Nano Lett.* **2021**, *21*, 1392−1399.
4. H. Wu, Q. Wang, A. Zhang, G. Niu, M. Nikl, C. Ming, J. Zhu, Z. Zhou, Y. Sun, G. Nan, G. Ren, Y. Wu, and J. Tang, *Sci. Adv.* **2023**, *9*, eadh1789.
5. X. Hu, P. Yan, P. Ran, L. Lu, J. Leng, Y. M. Yang, and X. Li, *J. Phys. Chem. Lett.* **2022**, *13*, 2862−2870.
6. M. Zhang, X. Wang, B. Yang, J. Zhu, G. Niu, H. Wu, L. Yin, X. Du, M. Niu, Y. Ge, Q. Xie, Y. Yan, and J. Tang, *Adv. Funct. Mater.* **2021**, *31*, 2007921.
7. M. Bilal, K. Zhou, T. He, S. Lin, A. Uddin, J. Yin, Q. He, O. F. Mohammed, and J. Pan, *Adv. Funct. Mater.* **2025**, e17266.
8. Q. Kong, X. Meng, S. Ji, Q. Wang, B. Yang, T. Bai, X. Wang, Z. Wang, R. Zhang, D. Zheng, F. Liu, and K. Han, *ACS Mater. Lett.* **2022**, *4*, 1734−1741.
9. J. H. Han, T. Samanta, Y. M. Park, H. J. Kim, N. S. Manikanta Viswanath, H. W. Kim, B. K. Cha, S. B. Cho, and W. B. Im, *ACS Energy Lett.* **2023**, *8*, 545−552.
10. Q. Hu, Z. Deng, M. Hu, A. Zhao, Y. Zhang, Z. Tan, G. Niu, H. Wu, and J. Tang, *Sci. China: Chem.* **2018**, *61*, 1581−1586.
